# Interference of Antinuclear Antibody (ANA) in Indirect Immunofluorescence Assay (IIFA)-Based Perinuclear Antineutrophil Cytoplasmic Antibody (pANCA) Interpretation

**DOI:** 10.1155/2022/1343805

**Published:** 2022-10-26

**Authors:** Sangeeta Deka, Deepjyoti Kalita, Udaykumar Sasi Rekha, Putul Mahanta, Diksha Rani, Ravi Shankar, Anusha Krishna Raj, Mithilesh Kumar Jha, Gaurav Badoni, Manisha Paul, Shailesh Kumar Gupta, Shailender Negi, Anshu Singh, Kuhu Chatterjee

**Affiliations:** ^1^All India Institute of Medical Sciences, Rishikesh, India; ^2^Assam Medical College, Dibrugarh, India

## Abstract

**Background:**

Indirect immunofluorescence assay (IIFA) based on antineutrophil cytoplasmic antibody (ANCA) testing is a commonly employed test for diagnosing autoimmune vasculitis. Antinuclear antibody (ANA) can give rise to a false interpretation of perinuclear-ANCA (pANCA) in ethanol-fixed granulocyte substrates. Analytical interference could frequently occur in setups where ethanol-fixed substrates are used alone. Here, we intend to investigate this ANA interference in pANCA interpretation.

**Methods:**

In this retrospective study, we studied anti-MPO-negative but ANA-positive and pANCA (IIFA based) samples. We also correlated immunoblot results (where data were available) and checked the association between grades of blot positivity (an indicator of the concentration of ANA) and frequency of pANCA interpretation. Data were analyzed by appropriate statistical techniques (Chi-square and kappa statistics).

**Results:**

About 19.2% of ANA blot (ENA-blot) positive samples displayed a pANCA positive pattern in the ethanol-fixed substrate, while this positivity in ENA-blot negatives was 6.5%. In positive ANA-IIFA samples, about 14.7% yielded pANCA patterns (on ethanol fixed substrates). Out of this, nuclear homogenous pattern yielding samples gave the highest frequency pANCA, that is, in 31.5% followed by speckled (11.1%), DFS (10.3%), and centromere (6.7%).The association of the nuclear homogenous pattern was statistically significant.

**Conclusions:**

ANA-positive results may interfere with the interpretation of pANCA as observed in ANA-IIFA and ENA-blot positive samples. ANA-IIFA patterns like nuclear homogenous may strongly associate this pANCA interpretation. This can help laboratories perform ANCA testing more effectively, ruling out ANA interference in ANCA screening.

## 1. Introduction

Antineutrophil cytoplasmic antibodies (ANCAs) are crucial in diagnosis and pathogenesis of a group of conditions called ANCA-associated vasculitis [1, 2]. ANCA-associated vasculitis is a rare disease associated with necrotizing inflammation of small/medium-sized blood vessels with and without granuloma formation in affected organs [2, 3]. The primary syndromes include granulomatosis with polyangiitis (GPA), microscopic polyangiitis (MPA), and eosinophilic GPA [2].

ANCAs are usually detectable in such conditions [2, 3]. In indirect immunofluorescence-based assays (IIFA), two common patterns of ANCAs are observed—cANCA (cytoplasmic ANCA) and pANCA (perinuclear-ANCA), representing two different antigen specificities, that is, proteinase 3 (PR3) and myeloperoxidase (MPO), respectively [4, 5]. Guideline for ANCA testing in small-vessel vasculitis advocates ELISA-based anti-MPO–anti-PR3 antibody detection by antigen-specific immunoassay as initial screening/evaluation of suspected cases [6]. ANCA-IIFA is reserved to confirm borderline/low positive or negative cases with high clinical suspicion. [6] But, consensus statement states that ANCA-IIFA is based on the notion that this is of higher sensitivity and lesser specificity than specific antibody-assays [6, 7]. Hence, the demand for IIFA-based tests (adjunct or confirmatory) is very high, and they are commonly performed tests.

IIFA-based ANCA detection test is not without its share of challenges. The presence of antibodies against antigens like elastase, cathepsin *G*, azurocidin, lactoferrin, lysozyme, and bactericidal/permeability-increasing factors can yield pANCA (or atypical pANCA) pattern [8, 9]. Antinuclear antibody (ANA) presence can be a reason for pANCA positivity in the absence of anti-MPO [10–13]. This is observed in ethanol-fixed neutrophils though it can be ruled out by using an additional formalin-fixed substrate [14,15]. Double substrate (ethanol fixed HEp-2 and neutrophil substrate) can help rule out the interference [15]. But, these additional substrates bring in extra expenditure, mainly for screening purposes in resource scant set up. Hence, a pragmatic approach to cut the cost could be the use of ANA testing in pANCA positive but anti-MPO negative (or if anti-MPO testing is not available) samples to rule out the interference [16].

We took up this study to analyze our population's level of interference of ANA positivity (by IIFA and immunoblot) in pANCA-positive samples (ethanol-fixed neutrophils).

## 2. Methods

To include in the study, we considered those samples sent for ANA (by IIFA) and ANCA (done by IIFA and ELISA both). Samples considered were those received for testing of ANA and ANCA from different departments (internal medicine, pediatrics, ophthalmology, rheumatology, obstetrics & gynaecology, ENT, dermatology, etc.,) of our tertiary care teaching hospital in a continuous manner. Data were obtained retrospectively from January 2017 to December 2021. In our lab, blood received in plain vials is processed for serum separation, and thus obtained sera were preserved at −20^0^C prior to testing.

ANA-IIFA positivity was the starting point of our analysis, followed by results of ANCA testing (IIFA and ELISA).

Testing for ANCA in our lab: sera were subjected to IIFA-based ANCA testing (IIFT : Granulocyte Mosaic 13, Euroimmun, Lübeck, Germany, Catalog# FA1201-1005-13) and monospecific Sandwich third-generation ELISAs for anti-PR3 (anti-PR3-hn-hr-ELISA(IgG), Euroimmun, Lübeck, Germany, Catalog# EA1201-9601-2G) and anti-MPO (anti MPO-ELISA-IgG, Euroimmun, Lübeck, Germany, Catalog# EA1211-9601-G) to detect the presence of ANCA. [1].

The IIFA-based ANCA diagnostic kit used ethanol-fixed buffy coat human neutrophils (along with additional formalin-fixed neutrophil substrate and HEp-2-neutrophil substrate fixed in ethanol). The patient sera were diluted in 1/10 proportion, added to the substrate wells, and then incubated. In case sera containing ANCA (IgG, IgM, and IgA) were attached to antigens on fixed neutrophils [1]. FITC (green) labeled antihuman antibodies were allowed to react and observed under the fluorescence microscope. A regularly distributed granular fluorescence over the entire cytoplasm of the granulocytes (other than nuclei) was noted as cANCA (cytoplasmic pattern).

In contrast, a smooth fluorescence wrapped around the granulocyte's cell nuclei was recorded as pANCA (perinuclear pattern). ELISA kits had reagent wells coated with a mixture of purified recombinant PR3 and native PR3 (in anti-PR3-hn-hrELISA, IgG) as well as purified-MPO (in anti-MPO-ELISA, IgG) antigens in two respective kits [1]. Prediluted samples were added to the wells. After a period of incubation, an enzyme-labeled antihuman IgG (enzyme conjugate) was added, catalyzing a colour reaction to detect bound antibodies. A chromogenic substrate was added to determine the extent of enzyme activity by measuring colour intensity by spectrometry. The cutoff for both tests (PR3 and MPO) was 20 relative units (RU)/mL [1].

Testing for ANA : Euroimmun IIFA-based kit (IIFT Mosaic: HEp-20-10/Liver (Monkey) test system, Euroimmun, Lübeck, Germany, Catalog# FA1512-1010-1) was utilized for ANA detection. A 1 : 100 ratio diluted serum (30 *μ*l) was applied to the substrates in a biochip as per kit instruction. After a brief incubation at room temperature, PBS-Tween is applied and fluorescein-labeled antihuman globulin is poured into each reaction field. The slide was further incubated and observed under a fluorescent microscope. Patterns like nuclear homogenous, speckled, centromere, and mitosis positive (spindle/centrosome) were recorded. Few samples which were further found to be tested by immunoblot assay were noted down. This testing was carried out using Euroimmun ENA immuno blot strip (Euroline ANA Profile 3 plus DFS70 (IgG), Euroimmun, Lübeck, Germany, Catalogue# DL 1590-6401-30G). This kit has extractable nuclear antigen targets like nRNP/Sm, Sm, SSA, Ro-52, SSB, Scl-70, PM-Scl, PCNA, Jo-1, CENP-B, dsDNA, DFS70, nucleosomes, histones, ribosomal protein-P, antimitochondrial antibodies (AMA-M2), and control. The intensity of blot strip reaction was analyzed using image analysis software provided by the kit manufacturer (EUROLineScan Ver. 3.4.30, Euroimmun, Lübeck, Germany). A grading protocol (for positive targets) mentioned in the kit insert was followed. Grading went like from ++++, +++, and ++, + to ± as intensity of band colour (corresponding to the decreasing amount of antibody in the sample) decreases. Euroline scan software calculates this grade from the intensity of band colour in positive immunoblot images. In case of multiple target positivity, highest graded positive target was recorded for analytical work.

Data generated were analyzed statistically using SPSS v.23.0 (IBM, NY, United States). McNemar's Chi-squared test was applied to check the association of different subgroups with the outcome (pANCA positivity). To assess the agreement between two main tests (ANA-IIFA and ENA-blot), we compared the binary outcomes of the two methods using the kappa statistics. A *p*-value of less than 0.05 was considered significant in all the statistical tests. All described procedures were part of our lab's routine testing flow, and data were analyzed in a retrospective manner [1].

## 3. Results

**Figure 1:**
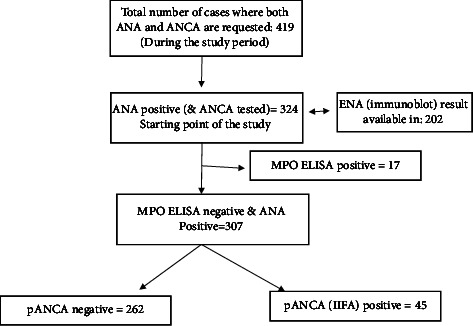
Flow diagram of the study.

A total of 324 ANA-positive cases (by ANA-IIFA and in selected cases also by immunoblot) undergoing ANCA (IIFA followed by PR3 and MPO ELISA) between January 2017 to December 2021 were included in this study. Out of these, anti-MPO positives (by ELISA) were excluded (*n* = 17) from the study (See Figure 1).

Table 1 depicts the ENA-blot records and pANCA interpretation in ethanol-fixed granulocyte substrate. About 6.5% of ENA-blot negative cases yielded a positive pANCA pattern. Overall, ENA-blot positive cases gave three times more positive apparent pANCA than ENA-blot negative cases (19.2% to 6.5%; *p*=0.045). No correlation could be observed between the semiquantitative ANA measurement (in terms of grades, i.e., ++++, +++,++,+ and +/-) and pANCA positivity, though statistically significant relation was observed in two grades (++ and +/-). This grading system is as per described in the product insert and calculated by using software [EUROLineScan Ver. 3.4.30, Euroimmun, Lübeck, Germany] from the scan images of blots. Association was significant between some ANA-IIFA patterns and apparent pANCA positivity, as shown in Table 2. In 31.5% of nuclear homogenous positive samples, pANCA was observed in a statistically significant way (*p*=0.0001). Speckled and DFS70 patterns were also linked to a higher proportion of pANCA records (Table 2) though the association was not statistically significant. Overall, pANCA positivity was about three times (14.7%) in ANA-IIFA-positive samples than in ANA-IIFA-negative samples (4.2%), and this was statistically significant (*p*=0.007) as depicted in Table 2. Cohen's kappa for two sets of tests (ANCA-IIFA in ENA-blot positive samples and ANCA-IIFA in ANA-IIFA samples) was satisfactory.

## 4. Discussion

In the current study, about 19.2% of ANA-positive (ENA-blot) subjects (postimmunoblot—see Table 1) showed pANCA interference. Similarly, the proportion of pANCA interference post-ANA-IIFA was found to be 14.7% (see Table 2). In immunoblot-based tests, the relative concentration of ANAs (represented by grade obtained upon analysis by EUROLineScan software, highest grade shown being considered, in case of multiple ANA target positivity in same sample) in a given sample was linked to frequency of pANCA interference (as shown in Table 1). It can be seen that interference at a lower grade (++) was more (statistically significant) than that in a higher grade (+++) of ANA positivity, which corroborates nicely with previous similar works [16]. The patterns of ANA (in IIFA) demonstrated good correlations with pANCA results. As depicted in Table 2, the nuclear homogenous pattern (AC1) is most frequently linked to possible pANCA interference (31.5% cases) compared to other patterns like speckled (11.1%), DFS70 (10.3%), and centromere (6.7%). Such pattern correlation tallies well with earlier works [16–18]. Some studies did not include pattern information, unlike ours [17]. We also analyzed immunoblot results (Table 1), which was unique about our current work.

ANCA-associated major antigens (myeloperoxidase and proteinase 3) are localized in cytoplasmic granules of the granulocytes [2, 8]. Upon fixation to ethanol, cells get dehydrated, accompanied by cellular membrane damage [2, 8]. MPO antigens are positively charged, get electrostatically attracted to DNA molecules (negatively charged) in the cells, and migrate towards the latter. This gives rise to the perinuclear pattern observable after the IIFA staining procedure give a pattern similar to pANCA in IIFA-ANCA testing (on the other hand, when the same substrate is fixed by formalin, granules and content are fixed in the cytoplasm itself, resulting in a cANCA pattern for both MPO and PR3) [2, 8]. Target antigens for anti-MPO antibody (MPO-antigen attracted electrostatistically towards DNA in an ethanol substrate) and antihistone/anti-dsDNA antibody (histone/dsDNA) are similar—this may explain why nuclear homogenous pattern (mainly due to antidouble-stranded DNA antibodies or antihistone antibodies) is more frequently prone to give pANCA interference (Table 2) [2, 3, 10, 18]. Ruling out ANA interference in a positive pANCA case scenario is critical as disease association, pathogenic mechanisms, clinical manifestation, and treatment options differ [3, 10].

Anti-MPO antibody presence in serum may be due to different types of epitopes present in MPO antigen. It is not possible to have a detection system (ELISA) to cover antibodies against all epitopes—some of these undetectable (by ELISA) may be clinically relevant (vasculitis causing) [3]. Hence, another possibility is that the pANCAs may be due to a variant of anti-MPO antibody specific for an epitope not included in the particular type of immunoassay/ELISA being undertaken or not available yet. Overall, the laboratory assays to differentiate a vasculitis-associated pANCA from pANCA with other antigen specificities are complex. A thorough understanding of the assay targets before a logical interpretation is essential [3].

Another area where the current work can be relevant is the testing strategy of a condition like autoimmune hepatitis. A nuclear homogenous ANA and atypical ANCA pattern are the critical laboratory markers for type 1 autoimmune hepatitis [19]. Latter is considered a selective marker of type 1 autoimmune hepatitis. As antibodies (pANCA-related) here are specific against the peripheral nuclear antigen and not against cytoplasmic antigens, it will show a positive pANCA pattern in ethanol-fixed substrate and negative in the formalin-fixed substrate. This is very useful in the identification of atypical pANCA. The nuclear homogenous ANA pattern goes with this [19, 20].

It is pertinent to mention here that in a majority of autoimmune hepatitis related ANA-IIFA-positive cases (2/3rd), the pattern observed is nuclear homogenous (HEp-2 substrate) while rest (1/3rd) is either speckled or nucleolar. The antigens recognized in these cases are single- and double-stranded DNA, nucleolar chromatin, histone, centromere, cyclin A, ribonuclear protein, and unknown (30% cases) [21].

Two limitations of our current study were a small sample size and biased cohort (as ANA-IIFA-positive samples were taken as the starting point in a retrospective manner). We tried to overcome these limitations by taking measures such as long study duration (5 years), performing few tests (immunoblot) on preserved samples, including samples from multiple disciplines/departments covering different age, gender, clinical spectrum etc., and data analysis and data collection by different people.

The overall analysis indicates the existence of ANA interference upon ANCA interpretation in our population. In setups performing IIFA-based ANCA tests probably need to adopt an economical workflow to rule out or minimize error due to the issue of ANA interference. Existing clinical/testing guidelines advise IIFA-based ANCA testing low antibody-positive samples or negative antibody samples in the presence of clinical features of AAV [6, 7]. Adding an ANA screening test (for interference detection) in these scenarios can be ideal and logical. This approach can be an economically viable alternative to expensive options like using additional substrates (additional formalin-fixed granulocytes and ethanol-fixed HEp-2-granulocyte combined) with every ANCA test.

## Figures and Tables

**Table 1:** ANA blot (ENA) results (with grading) versus pANCA positivity.

S. No	ANA-blot (ENA blot) result grade^*∗*^	No of cases	pANCA positive (%)	Cohen's kappa	*p*-Value (at significant level <0.05)
01	++++	3	1 (33.3)		0.4224
02	+++	23	5 (21.7)		0.4566
03	++	59	15 (25.4)		**0.0248**
04	+	34	8 (23.5)		0.2135
05	(+)/-	37	1 (2.7)		**0.0131**
	Positives (overall)	156	30 (19.2)	0.822	**0.0405**
	ANA-blot negative	46	3 (6.5)		

**Table 2:** ANA-IIFA pattern results versus pANCA positivity.

Sr no	ANA-IIFA pattern	No of cases	Positive pANCA (%)	Cohen's kappa	*p*-Value (at significant level <0.05)
01	Nuclear homogenous (AC1)	89	28 (31.5)		**0.00001**
02	Speckled (AC4/AC5)	81	9 (11.1)		0.293
03	Centromere (AC3)	45	3 (6.7)		0.101
04	Mitosis positive (spindle/centrosome) (AC24/AC25)	73	2 (2.7)		**0.001**
05	Dense fine speckled (DFS) 70 (AC2)	29	3 (10.3)		0.372
06	All positive	307	45 (14.7)	0.822	**0.007**

## Data Availability

All data are available with the manuscript itself.

## Abbreviation

ENA: Extractable nuclear antigens
AAV: ANCA-associated vasculitis
ANCA: Antineutrophil cytoplasmic antibody
ANA: Antinuclear antibody
IIFA: Indirect immunofluorescence assay
pANCA: Perinuclear ANCA
cANCA: Cytoplasmic ANCA.
